# Identifying Optimal Surgical Intervention-Based Chemotherapy for Gastric Cancer Patients With Liver Metastases

**DOI:** 10.3389/fonc.2021.675870

**Published:** 2021-11-29

**Authors:** Min Sun, Hangliang Ding, Zhiqiang Zhu, Shengsheng Wang, Xinsheng Gu, Lingyun Xia, Tian Li

**Affiliations:** ^1^ Department of General Surgery, Taihe Hospital, Hubei University of Medicine, Shiyan, China; ^2^ Department of Anesthesiology, Institute of Anesthesiology, Taihe Hospital, Hubei University of Medicine, Shiyan, China; ^3^ Hubei Key Laboratory of Embryonic Stem Cell Research, Taihe Hospital, Hubei University of Medicine, Shiyan, China; ^4^ Department of General Surgery, Xinchang Hospital Affiliated to Wenzhou Medical University, Wenzhou, China; ^5^ Department of Pediatrics, Zhongnan Hospital of Wuhan University, Wuhan, China; ^6^ College of Basic Medical Sciences, Hubei University of Medicine, Shiyan, China; ^7^ Department of Stomatology, Taihe Hospital, Hubei University of Medicine, Shiyan, China; ^8^ School of Basic Medicine, Fourth Military Medical University, Xi’an, China

**Keywords:** gastric cancer, liver metastasis, hepatectomy, interventional therapy, network analysis

## Abstract

**Background:**

This study aimed at evaluating the effects of surgical treatments-based chemotherapy in the treatment of gastric cancer with liver metastases (GCLM). It has not been established whether Liver-directed treatment (LDT) options such as hepatectomy and gastrectomy plus chemotherapy (HGCT), radiofrequency ablation and gastrectomy plus chemotherapy (RFAG), transarterial chemoembolization and gastrectomy plus chemotherapy (TACEG), gastrectomy plus chemotherapy (GCT) enhance the survival of GCLM patients.

**Methods:**

We performed systematic literature searches in PubMed, EMBASE, and Cochrane library from inception to September 2021. We created a network plot to comprehensively analyze the direct and indirect evidence, based on a frequentist method. A contribution plot was used to determine inconsistencies, a forest plot was used to evaluate therapeutic effects, the publication bias was controlled by funnel plot, while the value of surface under the cumulative ranking curves (SUCRA) was calculated to estimate rank probability.

**Results:**

A total of 23 retrospective studies were identified, involving 5472 GCLM patients. For OS and 1-, 2-, 3-year survival rate of all trials, meta-analysis of the direct comparisons showed significant better for HGCT treatments compared with GCT or PCT. In the comparison of the 5 treatments for 1-, 2-, 3-year survival rate, HGCT and RFAG were found to be more effective than GCT and PCT, respectively. By OS and 2-, 3-year survival rate analysis, RFAG was identified as the best option, followed by HGCT, TACEG, GCT and PCT. By 1-year survival rate analysis, HGCT and RFAG were identified as the most effective options.

**Conclusion:**

HGCT and RFAG has remarkable survival benefits for GCLM patients when compared to TACEG, GCT and PCT. HGCT was found to exhibit superior therapeutic effects for GCLM patients for 1-year survival rate while RFAG was found to be a prospective therapeutic alternative for OS and 2-, 3-year survival rate.

**Systematic Review Registration:**

identifier [10.37766/inplasy2020.12.0009].

## Introduction

Globally, gastric cancer is the fourth most common malignant tumor and the second highest cause of cancer-related mortalities (1–3). Therapeutic options for advanced gastric cancers have been enormously improved. In the last two decades, the 5-year survival rate is up to 40%. However, gastric cancer with liver metastases is considered a late-stage disease. Systemic chemotherapy was recommended as standard cure, with a 5-year survival rate of less than 10% (4, 5). The current standard management of GCLM is systemic chemotherapy with supportive care. Liver metastasis is a common phenomenon for many types of cancer (6–8). Liver-directed treatment (LDT) options such as hepatectomy and gastrectomy plus chemotherapy (HGCT), radiofrequency ablation and gastrectomy plus chemotherapy (RFAG), transarterial chemoembolization and gastrectomy plus chemotherapy (TACEG), gastrectomy plus chemotherapy (GCT) for GCLM is controversial (5, 9, 10). Compared to systemic chemotherapy, surgical treatment such as HGCT and RFAG of hepatic metastases presents favorable prognosis (11–13). According to the guidelines of The Committee of the Japan Gastric Cancer Association (JGCA) and National Comprehensive Cancer Network (NCCN), palliative management is recommended for stage IV gastric cancer, e.g. GCLM. In contrast, colorectal liver metastases are considered as suitable targets for radical surgery because they often present as liver-only metastatic disease, and R0 resection shows good prognostic outcomes, with a 5-year survival rate > 50% (14, 15). Retrospective studies have presented that the combination of hepatectomy and gastrectomy has visible survival outcome superiority (16–21). In the last two decades, along with the results of reported studies which demonstrated that radical surgery of primary gastric cancer and metastatic liver lesions had survival benefits, the Guidelines Committee of JGCA reconsidered the effect of surgical treatment in GCLM patients (22). Therefore, the role of LDT for GCLM is gradually being considered.

Previous therapeutic options for GCLM were HGCT, RFAG, TACEG, GCT and palliative chemotherapy (PCT). There are no randomized controlled clinical trials for GCLM therapies. In the present literature, majority of the studies are retrospective studies, which were performed at a single center, with a limited number of patients. Although some studies have confirmed the superior therapeutic outcomes of LDT, the clinical pathological characteristics of the involved patients reveal some selection bias, therefore, their results are difficult to accept. We performed a network meta-analysis to evaluate the survival benefits of LDT and systemic chemotherapy in the treatment of GCLM.

## Methods

### Study Protocol

This work was performed in accordance with the Preferred Reporting Items for Systematic Reviews and Meta-Analyses (PRISMA) of the C*ochrane Handbook for Systematic Reviews of Intervention* (23). The full protocol was registered and available on INPLASY (INPLASY2020120009).

### Search Strategy

We retrieved literature published in between 1966 and *September* 1st, 2021 by searching PubMed, EMBASE, and Cochrane Library with the keywords (1) “stomach neoplasm” OR “gastric neoplasms” OR “cancer of stomach” OR “stomach cancers” OR “gastric cancer” AND (2) “liver metastases OR liver metastasis OR hepatic metastasis” AND (3) “operative surgical procedure” OR ablation OR liver resection OR hepatectomy OR gastrectomy OR chemotherapy OR “interventional therapy” and using the search strategies as illustrated in **Supplementary Table 1**. We selected and evaluated all relevant studies and review articles about GCLM and inquired the authors for unpublished raw data. Searches were limited to English-language publications. In addition, the reference lists of the retrieved articles were examined for potential eligible studies.

### Study Selection

The inclusion criteria for the studies were: i. Systemic chemotherapy and surgical treatment; ii. Series of case control or cohort studies; iii. The number of patients were to be > 20; iv. Consists of available endpoints, such as overall survival, 1-, 2-, 3-, and 5-year survival rates, median survival time, and postoperative complications. The exclusion criteria for the studies were: i. studies with insufficient data or no related endpoints; ii. Missing control group.

### Data Extraction

Two researchers (MS and ZZ) independently extracted results from the enrolled articles in a standardized form. In addition, a third researcher (TL) was consulted in case there were disagreements. The information extracted from each study included the first author, country, year of publication, number of cases, treatment, sex, median or mean age of patients, study design, follow-up, median survival time. If a study did not report the Hazard Ratio (HR) of overall survival, we estimated HR and their corresponding 95% confidence intervals (CIs) using the method described by Parmar et al. (24) and Tierney et al. (25). We recovered the data of Kaplan-Meier curves as recently described by us (26, 27).

### Quality Assessment

We used the Newcastle-Ottawa Scale (NOS) to assess the quality of each included study. Scores ≥ 7 were considered high quality. We used a “star system” for case-control studies (**Supplementary Table 2**).

### Publication Bias

The funnel plots were used to establish publication bias. The funnel plot that was symmetrical near zero represented no publication bias.

### Statistical Analysis

The primary endpoint of this network meta-analysis (NMA) was overall survival (OS), defined as the time from random assignment to date of death from any cause or date of last follow-up. Secondary endpoints were 1-, 2-, 3-year survival rates. A pair-wise meta-analysis was performed by STATA 13.0 (Stata Corp, College Station, TX). R-3.6.3 and R packages gemtc were applied to conduct the Bayesian NMA, and 95% confidence intervals (CIs) were computed for HR in overall survival analysis. 1-, 2-, 3-year survival rates were analyzed while Odds Ratios (OR) with 95% confidence intervals (CIs) was calculated by fixed-effects or random-effects model (28, 29). Z test was performed to evaluate the significance of overall effect size.

A network plot was then used to directly demonstrate the whole information of included studies (30). Depending on direct comparison and indirect comparison outcomes, we estimated the contribution of each direct treatment comparison in the whole network structure, which was presented in a contribution plot. The inconsistency factor (IF) was calculated to determine the possible inconsistency in network comparison. The 95% CIs of IF values close to zero or the p value of Z test higher than 0.05 demonstrated there being no statistically significant inconsistency (31). Summary effects and corresponding predictive intervals were used to conclude relative mean effects and impact of heterogeneity in the network forest plot.

Finally, we calculated the surface under the cumulative ranking curve (SUCRA) of each treatment, which transformed the relative effects to the probability (2). SUCRA values range from 0 to 100%. The treatment was more valuable if the SUCRA value was higher. According to the estimated probability value, the treatments were ranked, which showed the percentage of effectiveness a treatment achieves with reference to an imaginary ideal treatment. Small-study effects was adjusted by a model of network meta-regression, the variance of the log-odds ratios as covariation (32).

## Results

### Study Characteristics

**Figure 1:**
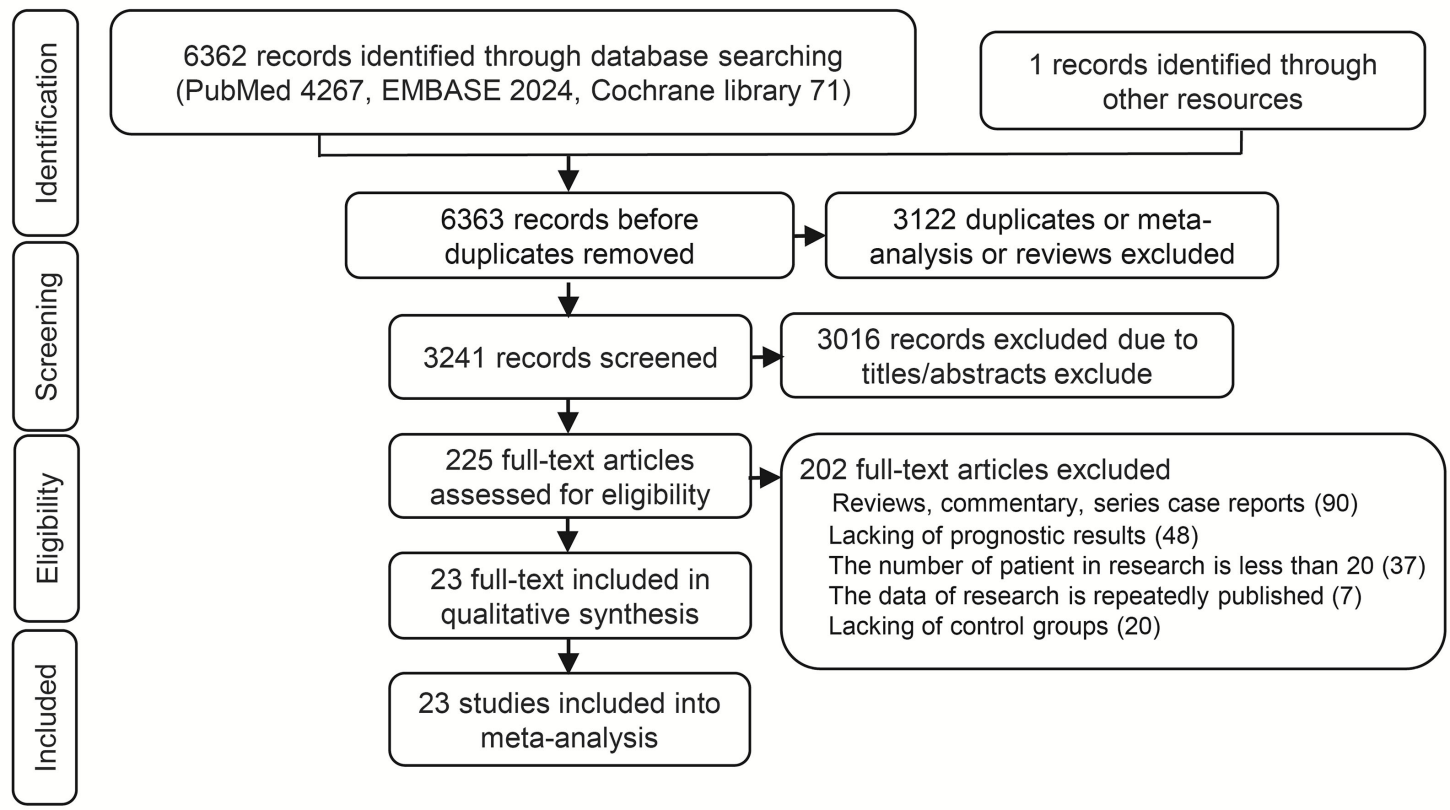
Flow chart of the identification process for eligible studies.

A total 6362 relevant articles were downloaded. The flow diagram documenting the search and inclusion of relevant studies is displayed in **Figure 1**. After considering the inclusion and exclusion criteria, a total of 23 retrospective studies involving 5472 GCLM patients were identified (12, 33–57). At least one of the following treatments were assessed by the study: HGCT, RFAG, GCT, PCT, and TACEG. Eight studies were three-arm trials while fifteen studies were two-arm trials. Further characteristics and Newcastle-Ottawa scale results regarding the included studies are presented in **Table 1** (**Supplementary Table 2**).

**Table 1:** The major clinical and survival information of included eighteen studies.

Study	Author	Country	Year	Number of Patients	Median age (years)	Follow up (months)	Hepatectomy arm	Synchronous	LVI	G3	Unilobar	Solitary	T3-4	R0	N+	Median survival time (months)	Tumor size of hepatic metastasis (cm)	Tumor size of gastric cancer (cm)	NOS score
1	Markar (37)	Japan	2016	217	65	8.3	Minor liver resections	78	63	NA	217	NA	NA	NA	NA	NA	NA	NA	9
2	Guner (12)	Japan	2016	98	NA	NA	NA	39	49	NA	64	NA	72	NA	78	HGCT 24; RFAG 23	NA	NA	7
3	Guan (33)	China	2016	136	63	NA	Partial hepatectomy	71	NA	NA	62	136	NA	NA	NA	PCT 8.7; RFAG 10.1; GCT 13.3	NA	NA	8
4	Yao (38)	China	2015	49	NA	NA	Irregular hepatectomy	NA	NA	NA	34	NA	NA	NA	NA	HGCT 24; GCT 12	NA	NA	8
5	Shinohara (39)	Japan	2015	47	66.7	NA	Liver resection	28	NA	16	25	18	25	NA	41	HGCT 22; GCT 7	NA	NA	8
6	Ohkura (40)	Japan	2015	34	67.2	22.4	NA	34	29	NA	NA	NA	24	NA	NA	NA	NA	NA	8
7	Liu (41)	China	2015	107	59 ± 1.7	NA	NA	107	NA	76	18	NA	NA	NA	NA	RFAG 5; PCT 3	NA	NA	8
8	Li (42)	China	2015	49	61.4 ± 9.5	19.6	NA	49	NA	18	NA	NA	39	NA	NA	GCT 20.5; PCT 9.1	NA	NA	9
9	Wang (58)	China	2014	66	61	14	Radical surgeries	66	NA	6	34	NA	31	NA	33	NA	NA	NA	7
10	Tiberio (43)	Italy	2014	195	68	NA	Hepatectomy	NA	NA	NA	NA	NA	NA	53	NA	HGCT 13; GCT 6.6; PCT 3	NA	NA	8
11	Chen (45)	China	2013	114	54	NA	Major hepatectomy	NA	NA	23	63	51	78	NA	17	HGCT 22.3; PCT 5.5	NA	NA	7
12	Miki (46)	Japan	2012	50	70	33.4	Hepatectomy	41	NA	NA	25	20	40	NA	NA	HGCT 33.4; GCT 10.5; PCT 8.7	NA	NA	8
13	Makino (47)	Japan	2010	63	65.8	16	Hepatectomy	31	NA	NA	30	24	NA	NA	54	NA	NA	NA	9
14	Lu (36)	China	2010	60	NA	NA	Hepatectomy	NA	NA	NA	34	34	NA	NA	NA	HGCT 20; RFAG 18; GCT 16	NA	NA	7
15	Kim (36)	Korea	2010	29	57.9	14.4	NA	12	NA	11	23	NA	21	NA	NA	RFAG 30.7; GCT 6.8	RFAG 2.8 ± 1.4; GCT 4.5 ± 1.5	RFAG 5.1± 2.3; GCT 6.1 ± 2.2	7
16	Cheon (48)	Korea	2008	58	61	15.5	Hepatectomy	42	NA	23	42	29	NA	NA	8	HGCT 21.7; RFAG 17; GCT 8.1	HGCT 2.4 ± 1.7	HGCT 5.7 ± 2.4	8
																	RFAG 2.1 ± 1.4	RFAG 6.6 ± 3.1	
																		GCT 6.1 ± 2.3	
17	Li (35)	China	2006	44	NA	NA	Hepatectomy	NA	NA	31	NA	NA	NA	NA	NA	HGCT 19.5; GCT 11; PCT 6.2	NA	NA	7
18	Li (49)	China, Taiwan	2017	653	68.28 ± 12.87	33	NA	NA	NA	NA	NA	NA	NA	NA	NA	GCT 3.13; HGCT 26.16	NA	NA	9
19	Shirasu (51)	Japan	2018	24	64.6	47.9	Partial hepatectomy	16	NA	5	10	2	NA	9	1	HGCT 24.8; PCT 38.1	NA	NA	9
20	Jagric (54)	Slovenia	2020	42	65.2 ± 8.49	NA	Metastasectomy	42	NA	19	23	23	40	18	NA	HGCT 9.3; GCT 4.2	NA	NA	9
21	Picado (57)	USA	2018	3175	64	21(10–32)	NA	42	NA	2168	NA	NA	260	137	1496	GCT 16; PCT 9.7	NA	NA	9
22	Tang (55)	China	2020	30	62	60	NA	35	18	33	36	31	46	NA	37	HGCT 21; RFAG 32; GCT 17	HGCT, 2.9 ± 1.6	NA	9
																	RFAG, 2.8 ± 1.7		
																	GCT, 2.1 ± 2.0		
23	Yu (56)	China	2020	132	62.5(32-75)	37.1(1-96)	NA	132	NA	46	36	NA	121	39	111	HGCT 33.6(26.6-40.6); PCT 12.4(10.0-14.8)	NA	NA	9

NA, Not available; Synchronous, Number of patients with synchronous liver metastases; N+, Number of patients with lymph-node involvement of the primary cancer; T3-4, Number of patients with stage pT3 or pT4; LVI, Number of patients with lymphovascular involvement; G3, Number of patients with G3 primary cancer; Unilobar, Number of patients with unilobar liver involvement; R0, Number of patients who achieved an R0 surgical removal on both primary cancer and liver metastases; Solitary, Number of patients with solitary liver metastases; HGCT, hepatectomy and gastrectomy plus chemotherapy; GCT, gastrectomy plus chemotherapy; PCT, palliative chemotherapy; RFAG, radiofrequency ablation and gastrectomy plus chemotherapy; TACEG, transarterial chemoembolization and gastrectomy plus chemotherapy.

### Direct Comparisons and Subgroup Analysis

For OS and 1-, 2-, 3-year survival rate of all trials, meta-analysis of the direct comparisons showed significant better for HGCT treatments compared with GCT or PCT, with the exception of RFAG (**Table 2**). As to OS and 1-, 2-, 3-year survival rate of all trials, PCT predicted a significantly worse OS than GCT (**Table 2**). For 1-, 2-, 3-year survival rate, the results showed that RFAG indicated a better survival rate than GCT (**Table 2**). Analysis of Asian subgroups showed that HGCT were better than GCT in OS, and 1-, 2-, 3-year survival rate, RFAG were better than GCT in 1-, 2-, 3-year survival rate (**Table 2**). Overall, statistical heterogeneity was moderate, although for most comparisons 95% CIs were wide and included values indicating very high or no heterogeneity, which portrayed the small number of studies available for every pair-wise comparison. In the meta-analyses of direct comparisons for OS and 1-, 2-, 3-year survival rate, I² values higher than 40% were recorded for the comparisons HGCT versus GCT and HGCT versus PCT (**Table 2**).

**Table 2:** Summary estimates for 1-, 2-, 3-year survival rates in meta-analyses of direct comparisons between pairs of Liver-directed treatment and subgroup analysis of Asian for GCLM.

Outcome	Subgroups	No. of trials	OR, FEM	95%CI, FEM	P value of FEM	OR, REM	95%CI, REM	P value of REM	I^2^	Heterogeneity P
All trials										
OS	GCT *vs* HGCT	12	2.147	[1.819; 2.534]	<0.0001	**2.209**	**[1.744; 2.797]**	**<0.0001**	41.30%	0.066
	PCT *vs* HGCT	8	2.797	[2.3; 3.402]	<0.0001	**2.664**	**[1.991; 3.563]**	**<0.0001**	47.30%	0.065
	RFAG *vs* HGCT	3	1.07	[0.725; 1.580]	0.734	1.07	[0.725; 1.580]	0.734	0.00%	0.81
	GCT *vs* PCT	2	**0.551**	**[0.467; 0.650]**	**<0.0001**	0.551	[0.467; 0.650]	<0.0001	0.00%	0.418
1-year survival rates	HGCT *vs* GCT	13	4.173	[3.090; 5.635]	<0.0001	**4.438**	**[2.852; 6.905]**	**<0.0001**	43.79%	0.0455
	HGCT *vs* PCT	8	5.831	[3.957; 8.591]	<0.0001	**5.765**	**[3.286; 10.113]**	**<0.0001**	44.09%	0.0847
	HGCT *vs* RFAG	3	1.084	[0.538; 2.186]	0.8207	1.091	[0.540; 2.203]	0.8075	0.00%	0.6299
	HGCT *vs* TACEG	1	0.816	[0.211; 3.159]	0.7688	0.816	[0.211; 3.159]	0.7688	NA	<0.0001
	GCT *vs* PCT	7	**2.957**	**[2.308; 3.788]**	**<0.0001**	2.944	[2.297; 3.775]	<0.0001	0.00%	0.7182
	GCT *vs* RFAG	3	**0.248**	**[0.100; 0.617]**	**0.0027**	0.25	[0.099; 0.633]	0.0035	0.00%	0.5505
	GCT *vs* TACEG	2	1.103	[0.559; 2.174]	0.7779	0.688	[0.055; 8.540]	0.7711	89.22%	0.0023
	PCT *vs* TACEG	2	0.34	[0.186; 0.622]	0.0005	0.322	[0.086; 1.206]	0.0925	78.12%	0.0325
2-year survival rates	HGCT *vs* GCT	8	5.311	[3.353; 8.410]	<0.0001	**5.076**	**[2.303; 11.185]**	**0.0001**	55.67%	0.0271
	HGCT *vs* PCT	6	4.707	[2.673; 8.289]	<0.0001	**4.824**	**[1.270; 18.330]**	**0.0209**	70.83%	0.0042
	HGCT *vs* RFAG	2	0.725	[0.296; 1.774]	0.4809	0.725	[0.296; 1.775]	0.481	0.00%	0.7939
	HGCT *vs* TACEG	1	0.468	[0.136; 1.611]	0.2284	0.468	[0.136; 1.611]	0.2284	NA	1
	GCT *vs* PCT	5	**3.106**	**[2.292; 4.209]**	**<0.0001**	3.059	[2.260; 4.140]	<0.0001	0.00%	0.9282
	GCT *vs* RFAG	3	**0.161**	**[0.050; 0.513]**	**0.002**	0.158	[0.026; 0.958]	0.0448	39.87%	0.1895
	GCT *vs* TACEG	2	0.702	[0.291; 1.691]	0.4303	0.488	[0.001; 238.526]	0.8202	91.40%	0.0006
	PCT *vs* TACEG	2	0.849	[0.238; 3.028]	0.801	0.732	[0.025; 21.063]	0.8555	77.22%	0.0361
3-year survival rates	HGCT *vs* GCT	11	**4.742**	**[2.699; 8.333]**	**<0.0001**	4.556	[2.574; 8.061]	<0.0001	0.00%	0.9186
	HGCT *vs* PCT	7	5.157	[2.628; 10.120]	<0.0001	**5.565**	**[1.811; 17.103]**	**0.0027**	48.73%	0.0689
	HGCT *vs* RFAG	3	0.877	[0.454; 1.695]	0.6967	0.877	[0.453; 1.698]	0.696	0.00%	0.7758
	HGCT *vs* TACEG	1	0.988	[0.248; 3.935]	0.9866	0.988	[0.248; 3.935]	0.9866	NA	1
	GCT *vs* PCT	5	**4.227**	**[2.822; 6.332]**	**<0.0001**	4.295	[2.908; 6.345]	<0.0001	0.00%	0.9903
	GCT *vs* RFAG	3	**0.153**	**[0.034; 0.687]**	**0.0144**	0.176	[0.037; 0.842]	0.0296	0.00%	0.5427
	GCT *vs* TACEG	2	0.332	[0.067; 1.645]	0.1768	0.454	[0.011; 19.365]	0.6799	65.83%	0.0871
	PCT *vs* TACEG	1	0.139	[0.006; 3.507]	0.2309	0.139	[0.006; 3.507]	0.2309	NA	<0.0001
Trials of Asian population										
OS	GCT *vs* HGCT	10	2.283	[1.861; 2.800]	<0.0001	**2.308**	[**1.695; 3.143**]	**<0.0001**	47.40%	0.047
	PCT *vs* HGCT	7	2.67	[2.142; 3.328]	<0.0001	**2.505**	[**1.770; 3.546**]	**<0.0001**	51.90%	0.052
	RFAG *vs* HGCT	3	1.07	[0.725; 1.580]	0.734	1.07	[0.725; 1.580]	0.734	0.00%	0.81
	GCT *vs* PCT	2	**0.503**	[**0.346; 0.732**]	**<0.0001**	0.503	[0.346; 0.732]	<0.0001	0.00%	0.526
1-year survival rates	HGCT *vs* GCT	10	**5.565**	**[3.716; 8.333]**	**<0.0001**	5.266	[3.192; 8.688]	<0.0001	26.94%	0.196
	HGCT *vs* PCT	6	**4.316**	**[2.685; 6.937]**	**<0.0001**	4.465	[2.240; 8.901]	<0.0001	43.24%	0.1169
	GCT *vs* PCT	4	**3.027**	**[1.675; 5.472]**	**0.0002**	3.034	[1.673; 5.500]	0.0003	0.00%	0.7838
	HGCT *vs* RFAG	3	1.084	[0.538; 2.186]	0.8207	1.091	[0.540; 2.203]	0.8075	0.00%	0.6299
	GCT *vs* RFAG	3	**0.248**	**[0.100; 0.617]**	**0.0027**	0.25	[0.099; 0.633]	0.0035	0.00%	0.5505
	HGCT *vs* TACEG	1	0.816	[0.211; 3.159]	0.7688	0.816	[0.211; 3.159]	0.7688	NA	<0.0001
	GCT *vs* TACEG	2	1.103	[0.559; 2.174]	0.7779	0.688	[0.055; 8.540]	0.7711	89.22%	0.0023
	PCT *vs* TACEG	2	0.34	[0.186; 0.622]	0.0005	0.322	[0.086; 1.206]	0.0925	78.12%	0.0325
2-year survival rates	HGCT *vs* GCT	6	6.362	[3.692; 10.963]	<0.0001	**5.681**	**[2.138; 15.100]**	**0.0005**	58.25%	0.0351
	HGCT *vs* PCT	5	4.396	[2.442; 7.912]	<0.0001	4.417	[0.914; 21.340]	0.0645	76.07%	0.0022
	GCT *vs* PCT	3	**3.708**	**[1.480; 9.289]**	**0.0052**	3.621	[1.428; 9.178]	0.0067	0.00%	0.7139
	HGCT *vs* RFAG	2	0.725	[0.296; 1.774]	0.4809	0.725	[0.296; 1.775]	0.481	0.00%	0.7939
	GCT *vs* RFAG	3	0.161	[0.050; 0.513]	0.002	**0.158**	**[0.026; 0.958]**	**0.0448**	39.87%	0.1895
	HGCT *vs* TACEG	1	0.468	[0.136; 1.611]	0.2284	0.468	[0.136; 1.611]	0.2284	NA	1
	GCT *vs* TACEG	2	0.702	[0.291; 1.691]	0.4303	0.488	[0.001; 238.526]	0.8202	91.40%	0.0006
	PCT *vs* TACEG	2	0.849	[0.238; 3.028]	0.801	0.732	[0.025; 21.063]	0.8555	77.22%	0.0361
3-year survival rates	HGCT *vs* GCT	9	**4.354**	**[2.355; 8.049]**	**<0.0001**	4.2	[2.255; 7.824]	<0.0001	0.00%	0.8549
	HGCT *vs* PCT	6	4.765	[2.379; 9.542]	<0.0001	**5.158**	**[1.474; 18.055]**	**0.0103**	55.95%	0.0449
	GCT *vs* PCT	3	3.764	[0.849; 16.686]	0.0811	3.751	[0.843; 16.685]	0.0826	0.00%	0.9585
	HGCT *vs* RFAG	3	0.877	[0.454; 1.695]	0.6967	0.877	[0.453; 1.698]	0.696	0.00%	0.7758
	GCT *vs* RFAG	3	**0.153**	**[0.034; 0.687]**	**0.0144**	0.176	[0.037; 0.842]	0.0296	0.00%	0.5427
	HGCT *vs* TACEG	1	0.988	[0.248; 3.935]	0.9866	0.988	[0.248; 3.935]	0.9866	NA	1
	GCT *vs* TACEG	2	0.332	[0.067; 1.645]	0.1768	0.454	[0.011; 19.365]	0.6799	65.83%	0.0871
	PCT *vs* TACEG	1	0.139	[0.006; 3.507]	0.2309	0.139	[0.006; 3.507]	0.2309	NA	<0.0001

OR, Odds ratios; CI, confidence interval; FEM, Fixed-effect Model; REM, Random-effect Model; NA, not available..

I^2^: index for assessing heterogeneity; value ≥40% indicates a moderate to high heterogeneity.

Bold indicate statistically significant values (P < 0.05).

### Network Meta-Analysis

**Figure 2:**
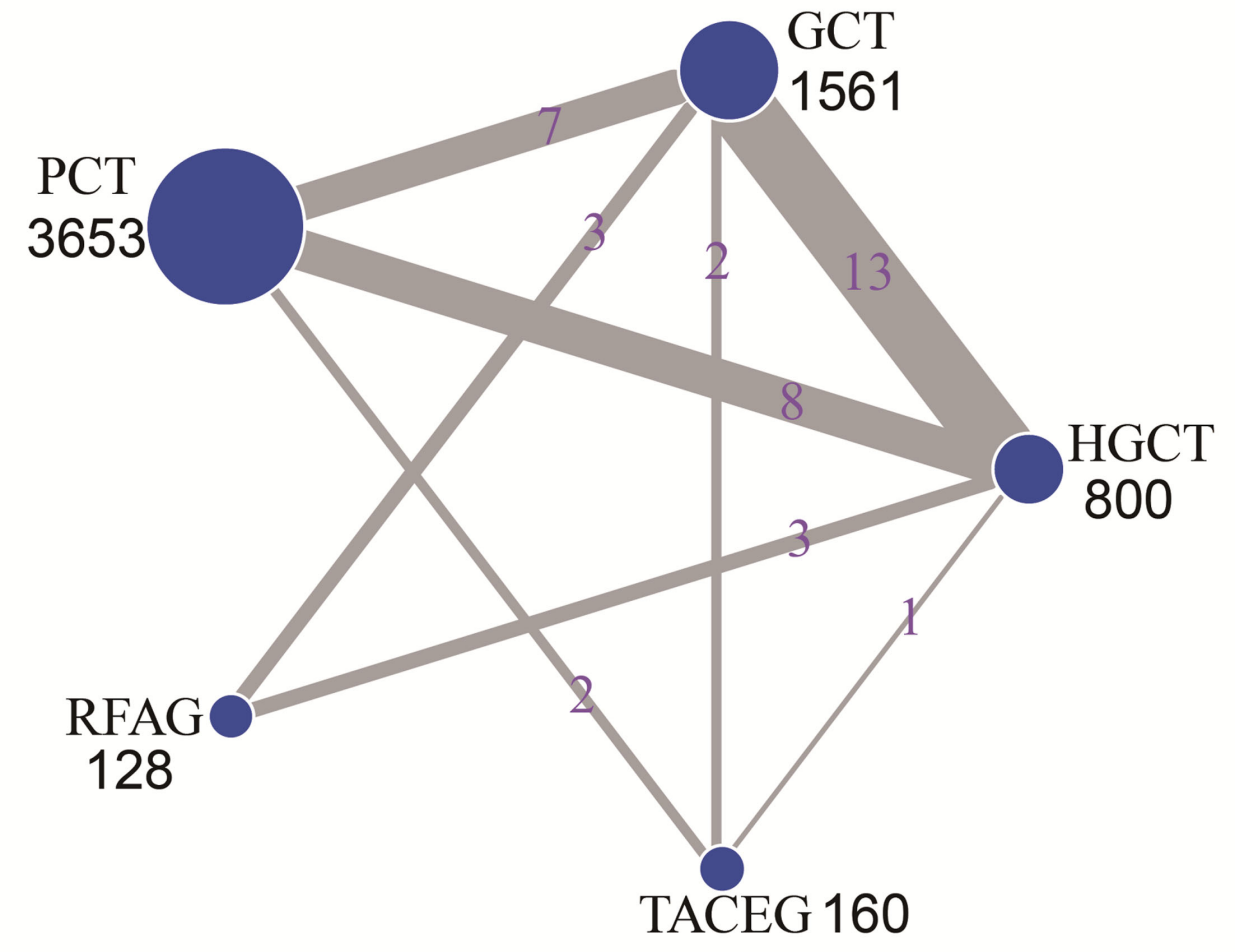
Network of eligible comparisons for the multiple-treatment meta-analysis. The size of each node represents the total sample size of treatment. The thickness of each line represents the total number of studies that compare each other. HGCT, hepatectomy and gastrectomy plus chemotherapy; GCT, gastrectomy plus chemotherapy; PCT, palliative chemotherapy; RFAG, radiofrequency ablation and gastrectomy plus chemotherapy; TACEG, transarterial chemoembolization and gastrectomy plus chemotherapy.

The network evidence plot is shown in **Figure 2**. Five treatments were included for analysis; HGCT, GCT, PCT, RFAG and TACEG, respectively. Comparing the studies with regards to their OS, 1-, 2-, 3-year survival rates, HGCT had the highest number of related studies and number of patients, while RFAG had the least number of patients and TACEG had the least number of related studies.

**Figure 3:**
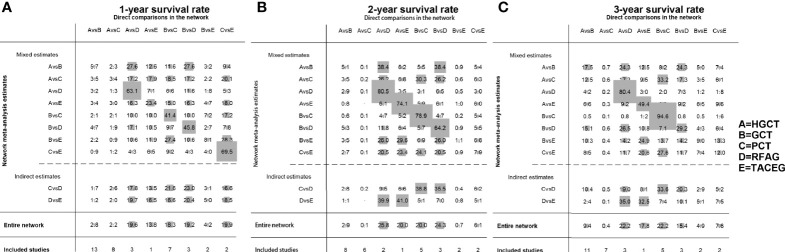
Contribution plot of the included studies. The columns refer to the direct comparisons and the rows refer to all possible pairwise comparisons. **(A)** Contribution plot for 1- year survival rate. **(B)** Contribution plot for 2- year survival rate. **(C)** Contribution plot for 3- year survival rate. HGCT, hepatectomy and gastrectomy plus chemotherapy; GCT, gastrectomy plus chemotherapy; PCT, palliative chemotherapy; RFAG, radiofrequency ablation and gastrectomy plus chemotherapy; TACEG, transarterial chemoembolization and gastrectomy plus chemotherapy.

The contribution plot is presented in **Figure 3**. Ten comparisons were made in the network analysis. All of them are mixed comparisons. In the overall contribution of network analysis, the remarkable influence evidence in the comparisons of 1-, 2-, 3-year survival rate is PCT vs. TACEG (19.9%), HGCT vs. RFAG (25.8%), GCT vs. PCT (22.2%), respectively.

There was no inconsistency between direct and indirect point estimates. In our network, there were 5 closed loops (**Supplementary Figure 1**). All confidence intervals for inconsistency factors (IFs) were compatible with zero inconsistency (IF=0) for all study outcomes (**Supplementary Figure 1**).

### Network Comparison

**Figure 4:**
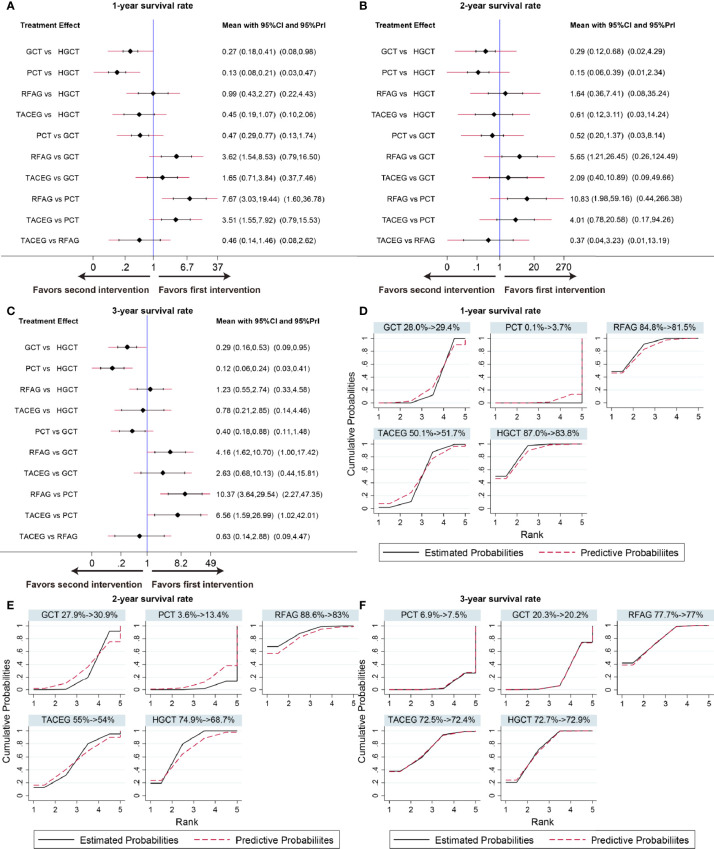
Confidence interval plot and SUCRA for the network analysis. The black and red solid lines represent the 95% confidence interval and the predictive intervals for summary relative risks for each comparison in the confidence interval plot. The blue line is the line of no effect (relative risk equal to 1). Confidence interval plot of 1-, 2- and 3- year survival rate **(A–C)**. SUCRA of 1-, 2- and 3- year survival rate **(D–F)**. Black solid lines correspond to the unadjusted model and red dashed lines to the adjusted for small effects model. Ranking indicates the probability to be the best treatment.

The summary effects with 95% CI are shown in **Figure 4**. In the comparison of the 5 treatments for 1-year survival rate, HGCT and RFAG were found to be more effective than GCT and PCT, respectively. GCT and TACEG was found to be more effective than PCT while there was no difference between HGCT and RFAG (**Figure 4A**). In the comparison of 2-year survival rates, HGCT and RFAG were found to be more effective than GCT and PCT, respectively. Other comparisons did not exhibit any significant differences (**Figure 4B**). In the comparison of 3-year survival rate, HGCT and RFAG were found to be more effective than GCT and PCT, respectively. GCT and TACEG was found to be more effective than PCT while there was no difference between HGCT and RFAG (**Figure 4C**).

### Ranking of Treatment

**Figures 4D–F** shows the relative ranking distribution of estimated cumulative probabilities for each treatment. The surface under the cumulative ranking curve (SUCRA) was adjusted by small-study effects. The SUCRA value rankings of 1-year survival rate were HGCT (83.8%), RFAG (81.5%), TACEG (51.7%), GCT (29.4%), and PCT (3.7%). The SUCRA value rankings of 2-year survival rate were RFAG (83%), HGCT (68.7%), TACEG (54%), GCT (30.9%), and PCT (13.4%). The SUCRA value rankings of 3-year survival rate were RFAG (77%), HGCT (72.9%), TACEG (72.4%), GCT (20.2%), and PCT (7.5%).

### Network Comparison, Ranking of Treatment and Subgroup Analysis of OS

**Figure 5:**
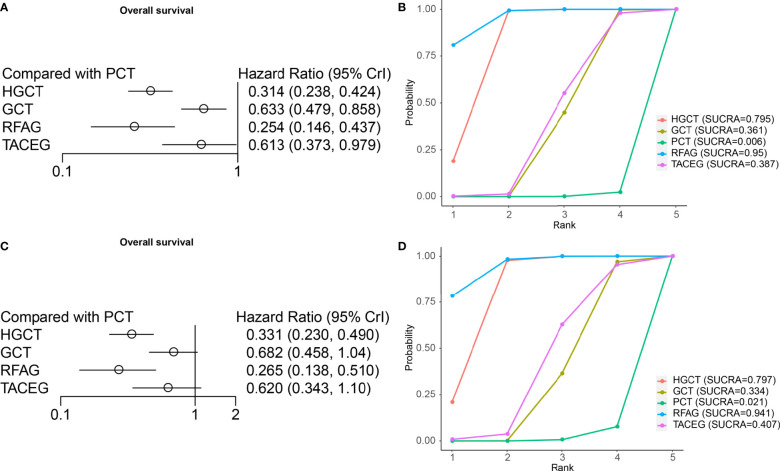
Results from network meta-analyses incorporating direct and indirect comparisons between the eligible interventions (OS). **(A)** Forest plot of each intervention versus PCT. **(B)** SUCRA plot of each intervention. **(C)** Forest plot of each intervention versus PCT in the Asian population. **(D)** SUCRA plot of each intervention in the Asian population.

On OS analysis, four treatments (HGCT, GCT, RFAG, TACEG) showed an HR in favor of OS (HR range, 0.146-0.979) (**Figure 5A**) in Asian and Caucasian. RFAG was identified as the best option, based again on HR and the SUCRA (**Figure 5B**), followed by HGCT, TACEG, GCT and PCT. In Asian population, HGCT and RFAG had the most favorable HR (HGCT HR, 0.331 [95% CI, 0.230-0.490; RFAG HR, 0.265 [95% CI, 0.138-0.510]) (**Figure 5C**); they ranked, on median, first and second in all the simulations (**Figure 5D**).

### Subgroup Analysis of Network Comparison in Asian Population

**Figure 6:**
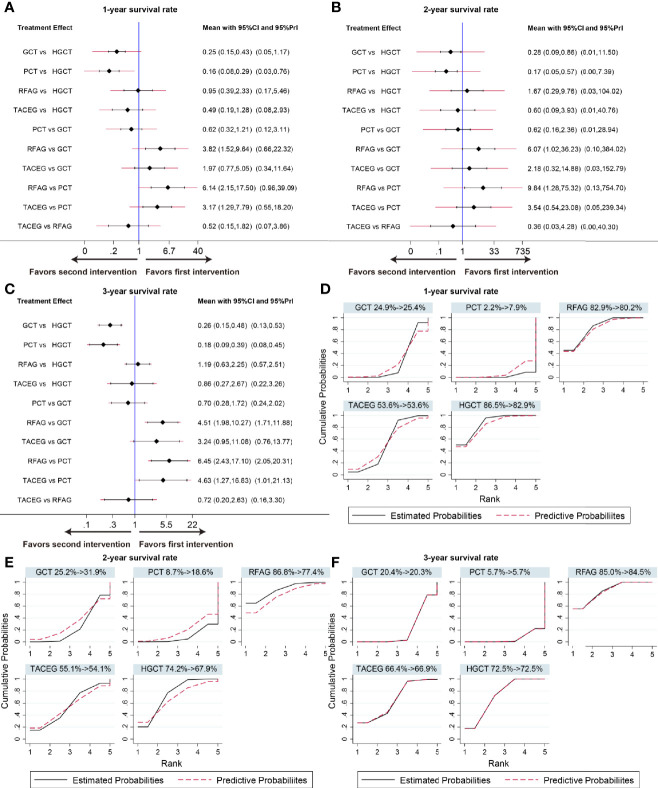
Confidence interval plot and SUCRA for the network analysis in the Asian population. The black and red solid lines represent the 95% confidence interval and the predictive intervals for summary relative risks for each comparison in the confidence interval plot. The blue line is the line of no effect (relative risk equal to 1). Confidence interval plot of 1-, 2- and 3- year survival rate in the Asian population **(A–C)**. SUCRA of 1-, 2- and 3- year survival rate **(D-F)** in the Asian population. Black solid lines correspond to the unadjusted model and red dashed lines to the adjusted for small effects model. Ranking indicates the probability to be the best treatment.

The summary effects in Asian population with 95% CI are shown in **Figure 6**. In the comparison of the 5 treatments for 1-, 3- year survival rate, HGCT and RFAG were found to be more effective than GCT and PCT, respectively. TACEG was found to be more effective than PCT while there was no difference between GCT and PCT (**Figures 6A, C**). In the comparison of 2-year survival rates, HGCT and RFAG were found to be more effective than GCT and PCT, respectively. Other comparisons did not exhibit any significant differences (**Figure 6B**). The SUCRA value rankings of 1-year survival rate were HGCT, RFAG, TACEG, GCT, and PCT (**Figure 6D**). The SUCRA value rankings of 2-, 3-year survival rate were RFAG, HGCT, TACEG, GCT, and PCT (**Figures 6E, F**).

### Publication Bias

**Figure 7:**
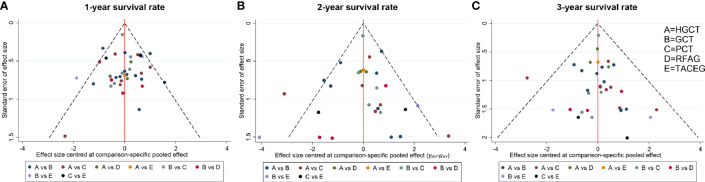
Comparison-adjusted funnel plot for the network meta-analysis. The red line suggests the null hypothesis that the study-specific effect sizes do not differ from the respective comparison-specific pooled effect estimates. Different colors represent different comparisons. **(A)** 1- year survival rate. **(B)** 2- year survival rate. **(C)** 3- year survival rate.

The funnel plot for network meta-analysis is presented in **Figure 7**. In general, all the selected studies were symmetrically distributed between the vertical line (x = 0). Therefore, there was no noteworthy publication bias in our network meta-analysis.

## Discussion

In this network meta-analysis, we revealed that HGCT and RFAG and has remarkable survival benefits for GCLM patients when compared to TACEG, GCT and PCT. By OS and 2-, 3-year survival rate analysis, RFAG was identified as the best option, followed by HGCT, TACEG, GCT and PCT. By 1-year survival rate analysis, HGCT and RFAG were identified as the most effective options. Due to non-specific symptoms, majority of gastric cancer patients were initially diagnosed with distant metastases. GCLM was considered as IV stage. The NCCN guideline recommends systemic chemotherapy as the standard cure for this group of patients. However, some controversies have been reported in the past two decades. Kim et al. (59) reported that gastrectomy or gastrectomy plus hepatectomy in GCLM patients has survival benefits when compared to chemotherapy. Tsujimoto et al. (60) showed that the 5-year survival rate of GCLM patients after hepatic resection was 31.5%, median survival time was 34 months. They also found that gastric tumor less than 6 cm and D2 lymphadenectomy were important factors for prognosis. Song’s study (61) suggested that surgical hepatic resection is beneficial for long-term survival in selected patients, with a 3-year survival rate of 47.6%. Groundbreaking by survival benefits of combined conversion therapy with surgery in patients with colorectal cancer liver metastases, numerous general surgeons navigated HGCT or RFAG in GCLM, which was thought over as a crucial strategy to alleviation disease and to prolong patient life (62–79). Liver-directed treatment (LDT) options for GCLM patients and surgical treatments were gradually attempted (12). If complete resection of liver metastases is possible, considering adequate hepatic reserve and surgical security, radical operations for primary gastric cancer and liver metastases lesions should be attempted (22, 79).

Considering the retrospective nature of the included studies and different selection biases for choosing patients on whom to perform radical surgery, their outcomes can hardly be regarded as a rationale in the treatment of GCLM, but it broadens the horizon of radical surgery in the selected GCLM patients. Furthermore, its prognostic value is considerable. Hepatic resection for liver metastases from colorectal cancer has been recommended as a standard treatment, 5-year survival was almost 40% (80). When the number of liver metastasis tumor ≤ 3, the diameter of single metastasis lesion ≤ 3 cm, the resection of primary gastric cancer and liver metastasis can also offer survival benefits in the GCLM patient (40). The security of surgical treatments for GCLM patients has also been confirmed. It does not enhance postoperative mortality (37). Studies also reported that GCLM patients with hepatectomy and gastrectomy exhibited favorable prognosis (2, 81–85). Therefore, we have confidence in the survival benefits of surgical hepatic resection. The value of this surgical treatment option is worth considering. According to the SUCRA values of the NMA, HGCT exhibited remarkable 1-, 2-, or 3-year survival and OS outcomes. Hepatic resection has survival superiority for selected GCLM patients.

RFAG was superior to the other therapies in 2-, or 3-year survival rate and OS analysis in this NMA. RFA has been thought over a less invasive therapeutic choice for GCLM (86). RFA can be used combined with systemic treatments (chemotherapy, targeted treatment, and immunotherapy), surgeries, and radiotherapy. RFAG which was radiofrequency ablation and gastrectomy plus chemotherapy showed comparable outcomes to curative resection (12, 48, 87). Cheon’s study suggested that a survival benefit of RFAG with curative intent was observed as compared with GCT, as evidenced by an improvement of 20.8% in the 5-year survival rate, corresponding to a 64.0% reduction in the risk of death (48). Kim et al. reported that The RFAG group showed a 76% decreased death rate compared to the GCT, was received well, and was found to be minor complications (34). Guner et al. suggested that in select patients with GCLM, HGCT and RFA showed satisfactory and comparable short- and long-term results, possible liver-directed treatment options for GCLM patients should be considered on an individual basis (12). Tang et al. suggested that OS were satisfactory and comparable between RFA and HGCT but better than those of chemotherapy, RFA is an appropriate option for patients with gastric cancer who have a solitary liver metastasis measuring ≤3.0 cm (55).

In our network analysis, we adopted several methods to control potential bias. First, the quality of all included studies was assessed by the Newcastle-Ottawa scale. The contribution plot was then performed to seek for significant bias in the network analysis. HGCT and RFAG exhibited the most impact on the 1-, 2- and 3-year survival rates, with 19.6%, 25.8% and 22.2% respectively, which was attributed to the small number of included patients. We also applied the small-study effects to adjust the value of SUCRA to control for potential bias. There was a low risk of publication bias.

Our study had some limitations. The retrospective nature of the included studies enhances the possibility of selection bias between different centers. Patient characteristics such as the number and size of hepatic metastasis, the location of metastasis lesions, the postoperative supportive treatment and adjuvant chemotherapy, which are vital prognostic factors to influence the survival benefits in GCLM patients could hardly ensure balance. However, it is difficult to perform prospective cohort studies for this group of patients die to the small number of GCLM patients in single centers and dismal prognosis with systemic chemotherapy. Our results recommend the HGCT or RFAG treatment option for GCLM patients when resection of gastric cancer and liver metastases lesions is feasible. This recommendation is in tandem with those of the EORTC and JCOG studies. Liver resection or RFA is a favorable option for GCLM patients without extrahepatic metastases, peritoneal dissemination and multiple hepatic metastases (22). Meanwhile, the maximum liver metastatic tumor size for which RFA is safe and effective remains highly controversial (55, 88).

To sum up, HGCT was found to exhibit superior therapeutic effects for GCLM patients while RFAG was found to be a prospective therapeutic alternative. Although we obtained data from retrospective studies, we confirmed the role of RFAG and HGCT as a therapeutic option for GCLM. Large-scale prospective studies in multiple centers are needed to further evaluate the survival benefits of potential radical surgery or RFAG in selected patients.

## Data Availability Statement

The original contributions presented in the study are included in the article/**Supplementary Material**. Further inquiries can be directed to the corresponding authors.

## Author Contributions

Conceptualization: TL and MS. Data curation: MS, LX, and ZZ. Formal analysis: SW and HD. Funding acquisition: XG, LX, and MS. Methodology: TL and MS. Project administration: TL and HD. Resources: MS and HD. Software: MS, ZZ, and SW. Supervision: TL and HD. Validation: TL. Visualization: MS and LX. Writing - original draft: MS, ZZ, and TL. Writing - review & editing: HD and TL. All authors contributed to the article and approved the submitted version.

## Funding

This research was supported by the National Natural Science Foundation of China (81902498), Hubei Provincial Natural Science Foundation (2019CFB450, 2019CFB177, 2016CFB530), Natural Science Foundation of Hubei Provincial Department of Education (Q20182105), Chen Xiao-ping Foundation for the Development of Science and Technology of Hubei Provincial (CXPJJH11800001-2018333), The Scientific and Technological Project of Shiyan City of Hubei Province (18Y35), The Foundation of Health and Family Planning Commission of Hubei Province (WJ2021Q007), and Innovation and Entrepreneurship Training Program (201810929005, 201810929009, 201810929068, 201813249010, S201910929009, S201910929045, S202013249005, S202013249008 and 202010929009).

## Conflict of Interest

The authors declare that the research was conducted in the absence of any commercial or financial relationships that could be construed as a potential conflict of interest.

## Publisher’s Note

All claims expressed in this article are solely those of the authors and do not necessarily represent those of their affiliated organizations, or those of the publisher, the editors and the reviewers. Any product that may be evaluated in this article, or claim that may be made by its manufacturer, is not guaranteed or endorsed by the publisher.

## Supplementary Material

Supplementary Figure 1 Inconsistency plot for the network meta-analysis. **(A)** inconsistency plot of 1- year survival rate. **(B)** inconsistency plot of 2- year survival rate. **(C)** inconsistency plot of 3- year survival rate.

## Abbreviations

GCLM, gastric cancer with liver metastasis; LDT, Liver-directed treatment; SUCRA, surface under the cumulative ranking curves; JGCA, Japan Gastric Cancer Association; NCCN, National Comprehensive Cancer Network; SCT, systemic chemotherapy; RoR, ratio of two odds ratios; IF, inconsistency factor; HGCT, hepatectomy and gastrectomy plus chemotherapy; GCT, gastrectomy plus chemotherapy; PCT, palliative chemotherapy; RFAG, radiofrequency ablation and gastrectomy plus chemotherapy; TACEG, transarterial chemoembolization and gastrectomy plus chemotherapy; HR, Hazard Ratio; NMA, network meta-analysis; OR, Odds ratio.
