# Supplementary figures and images for: Identifying Optimal Surgical Intervention-Based Chemotherapy for Gastric Cancer Patients With Liver Metastases
Source: Front Oncol. 2021 Nov 29;11:675870. doi: 10.3389/fonc.2021.675870 (PMC8666972; doi:10.3389/fonc.2021.675870)

**A****1-year survival rate**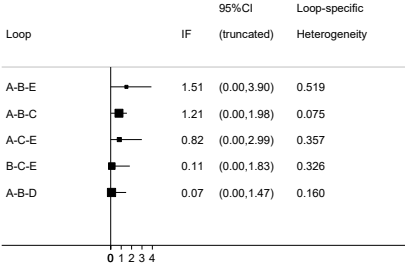**B****2-year survival rate**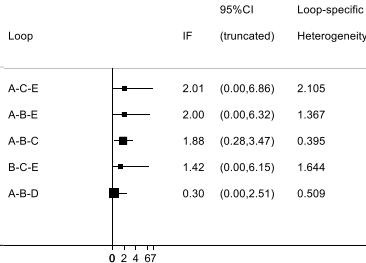**C****3-year survival rate**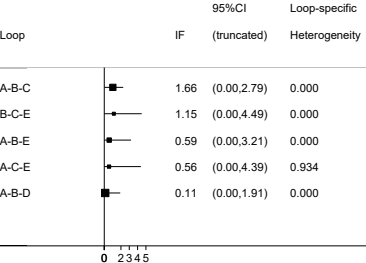

A=HGCT  
B=GCT  
C=PCT  
D=RFAG  
E=TACEG

Supplement: Supplementary Figure 1 — Inconsistency plot for the network meta-analysis. (A) inconsistency plot of 1- year survival rate. (B) inconsistency plot of 2- year survival rate. (C) inconsistency plot of 3- year survival rate. [file Image_1.pdf]
