# Supplementary material for: Identifying Optimal Surgical Intervention-Based Chemotherapy for Gastric Cancer Patients With Liver Metastases
Source: Front Oncol. 2021 Nov 29;11:675870. doi: 10.3389/fonc.2021.675870 (PMC8666972; doi:10.3389/fonc.2021.675870)
Supplement: Supplementary file 2 [file Table_1.docx]

**Supplementary Table 1. Search strategy for PubMed**

| **Database** | **No** | **PICOS** | **Strategy** | **Results** |
| --- | --- | --- | --- | --- |
| PubMed | #1 | P | (((((stomach[All Fields]) OR Gastric/)) AND (((((((((((Carcinoma/) OR malig*[tw]) OR neoplas*[tw]) OR oncol*[tw]) OR tumor/) OR Neoplasms/))) OR tumour/ OR cancer*/ OR Adenocarcinoma/))))) OR (((((((((Stomach Neoplasm/) OR Gastric Neoplasms/) OR Gastric Neoplasm/) OR "Cancer of Stomach"/) OR Stomach Cancers/) OR Gastric Cancer/) OR Gastric Cancers/) OR Stomach Cancer/) OR Cancer of the Stomach/) | 168,044 |
|  | #2 | P | liver metastases [TIAB] OR liver metastasis [TIAB] OR hepatic metastasis [TIAB] | 27,176 |
|  | #3 = #1 AND #2 | P | ((((((stomach[All Fields]) OR Gastric/)) AND (((((((((((Carcinoma/) OR malig*[tw]) OR neoplas*[tw]) OR oncol*[tw]) OR tumor/) OR Neoplasms/))) OR tumour/ OR cancer*/ OR Adenocarcinoma/))))) OR (((((((((Stomach Neoplasm/) OR Gastric Neoplasms/) OR Gastric Neoplasm/) OR "Cancer of Stomach"/) OR Stomach Cancers/) OR Gastric Cancer/) OR Gastric Cancers/) OR Stomach Cancer/) OR Cancer of the Stomach/)) AND (liver metastases [TIAB] OR liver metastasis [TIAB] OR hepatic metastasis [TIAB]) | 6288 |
|  | #4 | I | (Surgical Procedures, Operative[MeSH Terms]) OR ((((((((((((((Surgical operation[Title/Abstract]) OR Operation[Title/Abstract]) OR Surgery[Title/Abstract]) OR Ghost, Surgery[Title/Abstract]) OR Procedure, Operative Surgical[Title/Abstract]) OR Procedures, Operative[Title/Abstract]) OR Procedure, Operative[Transliterated Title]) OR Operative Procedure[Title/Abstract]) OR Operative Procedures[Title/Abstract]) OR Surgical Procedure, Operative[Title/Abstract]) OR Procedures, Operative Surgical[Title/Abstract]) OR Operative Surgical Procedures[Title/Abstract]) OR Operative Surgical Procedure[Title/Abstract])) | 3,900,033 |
|  | #5 | I | Ablation [TIAB] OR liver resection [TIAB] OR Hepatectomy [TIAB] OR gastrectomy [TIAB] OR Chemotherapy [TIAB] OR Interventional therapy [TIAB] | 523,769 |
|  | #6 = #4 OR #5 | I | ((Surgical Procedures, Operative[MeSH Terms]) OR ((((((((((((((Surgical operation[Title/Abstract]) OR Operation[Title/Abstract]) OR Surgery[Title/Abstract]) OR Ghost, Surgery[Title/Abstract]) OR Procedure, Operative Surgical[Title/Abstract]) OR Procedures, Operative[Title/Abstract]) OR Procedure, Operative[Transliterated Title]) OR Operative Procedure[Title/Abstract]) OR Operative Procedures[Title/Abstract]) OR Surgical Procedure, Operative[Title/Abstract]) OR Procedures, Operative Surgical[Title/Abstract]) OR Operative Surgical Procedures[Title/Abstract]) OR Operative Surgical Procedure[Title/Abstract]))) OR (Ablation [TIAB] OR liver resection [TIAB] OR Hepatectomy [TIAB] OR gastrectomy [TIAB] OR Chemotherapy [TIAB] OR Interventional therapy [TIAB]) | 4,213,289 |
|  | #7= #3 AND #6 | PI | (((((((stomach[All Fields]) OR Gastric/)) AND (((((((((((Carcinoma/) OR malig*[tw]) OR neoplas*[tw]) OR oncol*[tw]) OR tumor/) OR Neoplasms/))) OR tumour/ OR cancer*/ OR Adenocarcinoma/))))) OR (((((((((Stomach Neoplasm/) OR Gastric Neoplasms/) OR Gastric Neoplasm/) OR "Cancer of Stomach"/) OR Stomach Cancers/) OR Gastric Cancer/) OR Gastric Cancers/) OR Stomach Cancer/) OR Cancer of the Stomach/)) AND (liver metastases OR liver metastasis OR hepatic metastasis )) AND (((Surgical Procedures, Operative[MeSH Terms]) OR ((((((((((((((Surgical operation[Title/Abstract]) OR Operation[Title/Abstract]) OR Surgery[Title/Abstract]) OR Ghost, Surgery[Title/Abstract]) OR Procedure, Operative Surgical[Title/Abstract]) OR Procedures, Operative[Title/Abstract]) OR Procedure, Operative[Transliterated Title]) OR Operative Procedure[Title/Abstract]) OR Operative Procedures[Title/Abstract]) OR Surgical Procedure, Operative[Title/Abstract]) OR Procedures, Operative Surgical[Title/Abstract]) OR Operative Surgical Procedures[Title/Abstract]) OR Operative Surgical Procedure[Title/Abstract]))) OR (Ablation OR liver resection OR Hepatectomy OR gastrectomy OR Chemotherapy OR Interventional therapy )) | 4267 |
