# Supplementary material for: Identifying Optimal Surgical Intervention-Based Chemotherapy for Gastric Cancer Patients With Liver Metastases
Source: Front Oncol. 2021 Nov 29;11:675870. doi: 10.3389/fonc.2021.675870 (PMC8666972; doi:10.3389/fonc.2021.675870)
Supplement: Supplementary file 3 [file Table_2.docx]

**Supplementary Table 2. Results of quality assessment using the Newcastle–Ottawa Scale for the included studies.**

| **Study** | **Selection** |  |  |  | **Comparability** | **Exposure** |  |  | **Scores** |
| --- | --- | --- | --- | --- | --- | --- | --- | --- | --- |
|  | Is the case definition adequate? | Representativeness of the Cases | Selection of Controls | Definition of Controls | Comparability of Cases and Controls on the Basis of the Design or Analysis | Ascertainment of exposure | Same method of ascertainment for cases and controls | Non-Response rate | Total scores |
| Markar 2016 | ★ | ★ | ★ | ★ | ★★ | ★ | ★ | ★ | 9 |
| Guner 2016 | ★ | ★ | ★ | ★ | ★ | ★ | ★ | - | 7 |
| Guan2016 | ★ | ★ | ★ | ★ | ★★ | ★ | ★ | - | 8 |
| Yao 2015 | ★ | ★ | ★ | ★ | ★ | ★ | ★ | - | 8 |
| Shinohara 2015 | ★ | ★ | ★ | ★ | ★★ | ★ | ★ | - | 8 |
| Ohkura 2015 | ★ | - | ★ | ★ | ★★ | ★ | ★ | ★ | 8 |
| Liu 2015 | ★ | ★ | ★ | ★ | ★★ | ★ | ★ | - | 8 |
| Li 2015 | ★ | ★ | ★ | ★ | ★★ | ★ | ★ | ★ | 9 |
| Wang 2014 | ★ | ★ | - | ★ | ★ | ★ | ★ | ★ | 7 |
| Tiberio 2014 | ★ | ★ | ★ | ★ | ★★ | ★ | ★ | - | 8 |
| Chen 2013 | ★ | ★ | - | ★ | ★ | ★ | ★ | ★ | 7 |
| Miki 2012 | ★ | ★ | ★ | ★ | ★ | ★ | ★ | ★ | 8 |
| Makino 2010 | ★ | ★ | ★ | ★ | ★★ | ★ | ★ | ★ | 9 |
| Lu 2010 | ★ | ★ | ★ | ★ | ★ | ★ | ★ | - | 7 |
| Kim 2010 | ★ | ★ | - | ★ | ★ | ★ | ★ | ★ | 7 |
| Cheon 2008 | ★ | - | ★ | ★ | ★★ | ★ | ★ | ★ | 8 |
| Li 2006 | ★ | ★ | ★ | ★ | ★ | ★ | ★ | - | 7 |
| Li 2017 | ★ | ★ | ★ | ★ | ★★ | ★ | ★ | ★ | 9 |
| Shirasu 2018 | ★ | ★ | ★ | ★ | ★★ | ★ | ★ | ★ | 9 |
| Jagric 2020 | ★ | ★ | ★ | ★ | ★★ | ★ | ★ | ★ | 9 |
| Picado 2018 | ★ | ★ | ★ | ★ | ★★ | ★ | ★ | ★ | 9 |
| Tang 2020 | ★ | ★ | ★ | ★ | ★★ | ★ | ★ | ★ | 9 |
| Yu 2020 | ★ | ★ | ★ | ★ | ★★ | ★ | ★ | ★ | 9 |
