# Supplementary material for: Identifying Optimal Surgical Intervention-Based Chemotherapy for Gastric Cancer Patients With Liver Metastases
Source: Front Oncol. 2021 Nov 29;11:675870. doi: 10.3389/fonc.2021.675870 (PMC8666972; doi:10.3389/fonc.2021.675870)
Supplement: Supplementary file 4 [file Table_3.doc]

**MOOSE Checklist for Meta-analyses of Observational Studies**

**Associations between hepatectomy and survival of gastric cancer patients with liver metastases: A network analysis of 1441 patients**

| **Criteria** | | **Brief description of how the criteria were handled in the meta-analysis** |
| --- | --- | --- |
| **Reporting of background should include** | |  |
| √ | Problem definition | The current standard management of GCLM (gastric cancer patients with liver metastases) is systemic chemotherapy with supportive care. The application of hepatectomy combined with radical gastrectomy for GCLM is controversial. |
| √ | Hypothesis statement | Retrospective studies have presented that the combination of hepatectomy and gastrectomy has visible survival outcome superiority. Compared to systemic chemotherapy, surgical treatment of hepatic metastases presents favorable prognosis. In the last two decades, along with the results of reported studies which demonstrated that radical surgery of primary gastric cancer and metastatic liver lesions had survival benefits, the Guidelines Committee of JGCA reconsidered the effect of surgical treatment in GCLM patients.(13) Therefore, the role of liver resection for GCLM is gradually being considered. |
| √ | Description of study outcomes | 1- , 2- , 3- , and 5-year survival rates |
| √ | Type of exposure or intervention used | Previous therapeutic options for GCLM were systemic chemotherapy (CT), gastrectomy plus chemotherapy (G), hepatic interventional therapy plus gastrectomy (IT), and hepatectomy plus gastrectomy (HG). |
| √ | Type of study designs used | Series of case control or cohort studies |
| √ | Study population | The exclusion criteria for the studies were:  1) studies with insufficient data or no related endpoints;  2) Missing control group. |
| **Reporting of search strategy should include** | |  |
| √ | Qualifications of searchers | The credentials of the two investigators MS and ZZ are indicated in the author list. |
| √ | Search strategy, including time period included in the synthesis and keywords | We retrieved literature published in between 1966 and *November* 1st, 2020 by searching PubMed, EMBASE, and Cochrane Library.  See **Supplementary Table 1** in the Supplementary materials. |
| √ | Databases and registries searched | PubMed, EMBASE, and Cochrane Library |
| √ | Search software used, name and version, including special features | We did not employ a search software. EndNote was used to merge retrieved citations and eliminate duplications. |
| √ | Use of hand searching | We selected and evaluated all relevant studies and review articles about GCLM and inquired the authors for unpublished raw data. Searches were limited to English-language publications. In addition, the reference lists of the retrieved articles were examined for potential eligible studies. |
| √ | List of citations located and those excluded, including justifications | Details of the literature search process are outlined in **Supplementary Table 1.** |
| √ | Method of addressing articles published in languages other than English | Searches were limited to English-language publications. |
| √ | Method of handling abstracts and unpublished studies | We selected and evaluated all relevant studies and review articles about GCLM and inquired the authors for unpublished raw data.  We had contacted a few authors for published studies on the association. |
| √ | Description of any contact with authors | We selected and evaluated all relevant studies and review articles about GCLM and inquired the authors for unpublished raw data.  We contacted authors to find raw data regarding the mortality and cardiovascular effects via email. |
| **Reporting of methods should include** | |  |
| √ | Description of relevance or appropriateness of studies assembled for assessing the hypothesis to be tested | Detailed inclusion and exclusion criteria were described in the section of **Study selection**. |
| √ | Rationale for the selection and coding of data | Data extracted from each of the studies were relevant to the population characteristics, study design, exposure, outcome, and possible effect modifiers of the association. |
| √ | Assessment of confounding | The funnel plots were used to establish publication bias. The funnel plot that was symmetrical near zero represented no publication bias. |
| √ | Assessment of study quality, including blinding of quality assessors; stratification or regression on possible predictors of study results | We used the Newcastle-Ottawa Scale (NOS) to assess the quality of each included study. Scores ≥ 7 were considered high quality. We used a “star system” for case-control studies (Supplementary Table 2). |
| √ | Assessment of heterogeneity | The inconsistency factor (IF) was calculated to determine the possible inconsistency in network comparison. The 95% CIs of IF values close to zero or the p value of Z test higher than 0.05 demonstrated there being no statistically significant inconsistency. |
| √ | Description of statistical methods in sufficient detail to be replicated | Description of methods of meta-analyses and assessment of publication bias are detailed in the methods. |
| √ | Provision of appropriate tables and graphics | We included 1 box detailing the terms used for database search, 1 flow chart, 1 summary table, 1 forest plot of all studies. |
| **Reporting of results should include** | |  |
| √ | Graph summarizing individual study estimates and overall estimate | Fig 2-6, Fig S1 |
| √ | Table giving descriptive information for each study included | Table 1 |
| × | Results of sensitivity testing | None. |
| √ | Indication of statistical uncertainty of findings | 95% confidence intervals were presented with all summary estimates, IF values |
| **Reporting of discussion should include** | |  |
| √ | Quantitative assessment of bias | **Figure 6** |
| √ | Justification for exclusion | Fig 1 |
| √ | Assessment of quality of included studies | **Supplementary Table 2** |
| **Reporting of conclusions should include** | |  |
| √ | Consideration of alternative explanations for observed results | In this network meta-analysis, we revealed that HG has remarkable survival benefits for GCLM patients when compared to G and CT, the survival benefits between HG and IT did not exhibit any significant differences. Due to non-specific symptoms, majority of gastric cancer patients were initially diagnosed with distant metastases. GCLM was considered as IV stage. At six institutions in China, the role of surgery for GCLM patients was changed, and surgical treatments were gradually attempted. If complete resection of liver metastases is possible, considering adequate hepatic reserve and surgical security, radical operations for primary gastric cancer and liver metastases lesions should be attempted. |
| √ | Generalization of the conclusions | HG was found to exhibit superior therapeutic effects for GCLM patients while IT was found to be a prospective therapeutic alternative. |
| √ | Guidelines for future research | Although we obtained data from retrospective studies, we confirmed the role of HG as a therapeutic option for GCLM. |
| √ | Disclosure of funding source | This research was supported by the National Natural Science Foundation of China (81902498). |
